# Polyphonic sonification of electrocardiography signals for diagnosis of cardiac pathologies

**DOI:** 10.1038/srep44549

**Published:** 2017-03-20

**Authors:** Jakob Nikolas Kather, Thomas Hermann, Yannick Bukschat, Tilmann Kramer, Lothar R. Schad, Frank Gerrit Zöllner

**Affiliations:** 1 Computer Assisted Clinical Medicine, Medical Faculty Mannheim, Heidelberg University, Mannheim, Germany; 2 Department of Medical Oncology and Internal Medicine VI, National Center for Tumor Diseases, University Hospital Heidelberg, Heidelberg University, Heidelberg, Germany; 3 Ambient Intelligence Group, Center of Excellence in Cognitive Interaction Technology (CITEC), Bielefeld University, Bielefeld, Germany; 4 Klinik III für Innere Medizin, Herzzentrum der Universität zu Köln, Cologne, Germany

## Abstract

Electrocardiography (ECG) data are multidimensional temporal data with ubiquitous applications in the clinic. Conventionally, these data are presented visually. It is presently unclear to what degree data sonification (auditory display), can enable the detection of clinically relevant cardiac pathologies in ECG data. In this study, we introduce a method for polyphonic sonification of ECG data, whereby different ECG channels are simultaneously represented by sound of different pitch. We retrospectively applied this method to 12 samples from a publicly available ECG database. We and colleagues from our professional environment then analyzed these data in a blinded way. Based on these analyses, we found that the sonification technique can be intuitively understood after a short training session. On average, the correct classification rate for observers trained in cardiology was 78%, compared to 68% and 50% for observers not trained in cardiology or not trained in medicine at all, respectively. These values compare to an expected random guessing performance of 25%. Strikingly, 27% of all observers had a classification accuracy over 90%, indicating that sonification can be very successfully used by talented individuals. These findings can serve as a baseline for potential clinical applications of ECG sonification.

In medicine, technological advancements lead to a rapidly growing amount of data. Visualization techniques are usually applied to support data inspection and analysis^1^,^2^ However, visualization is just one way to make information intelligible to humans. An alternative approach is *sonification*, i.e. the systematic and reproducible representation of data by using sound^3^. Sonification has been applied to gene expression data^4^, DNA methylation data^5^, biological imaging data^6^, electroencephalography (EEG) signals^7^,^8^,^9^, electrocardiogram (ECG) signals^7^,^10^ and combinations of biomedical signals^11^.

Coming from a clinical background, we asked whether sonification techniques (used in so-called auditory displays) of complex data sets can aid clinicians in their diagnostic decision making. Specifically, we focused on ECG signals as complex multi-channel datasets with ubiquitous applications in the clinic. Although previous studies have proposed techniques for heart rate sonification^12^ and ECG sonification^10^,^11^, no study has evaluated in how far these techniques are actually suited for clinical application of ECG analysis.

To investigate this questions, we first designed a parameter-mapping sonification^13^ method that applies time-variant oscillators to convert the multi-channel ECG datasets into a polyphonic sound. Secondly, we applied this method to samples from a publicly available database^14^. Thirdly, we evaluated the diagnostic accuracy of common cardiac pathologies based on sonified ECG signals.

## Material and Methods

### Ethics statement

In this retrospective study, we used human ECG measurements that are openly accessible in a public database^14^. All patient data were fully anonymized and could not be traced back to any individual patient. Our institution’s medical ethics board II (Medical Faculty Mannheim, Heidelberg University, Germany) gave their consent to this data analysis (decision number 2016–856R-MA, granted to FGZ) and waived the need for informed consent by the respective patients. All analyses were carried out in accordance with the Declaration of Helsinki and in accordance with the ethics board approval.

### Dataset

We used a selection of 12-channel ECG signals from the “St.-Petersburg Institute of Cardiological Technics 12-lead Arrhythmia Database“ (incartdb) on www.physionet.org^14^. From the 12-channel datasets, we extracted the first six leads, corresponding to the electric vectors in the frontal plane (I, II, III, aVR, aVL, avF). We selected the following four pathologies: ST-elevation myocardial infarction (STEMI), premature ventricular contraction/ventricular extrasystole (PVC), atrial fibrillation and bigeminy. The reason for this selection was (a) that these pathological patterns represent frequent pathological findings in ECGs and (b) that these patterns were among the most frequent patterns in the database. For each category, we retrieved a single 10 s sample for training and three 10 s samples for testing. Furthermore, from the “PTB database”^15^,^16^ on www.physionet.org^14^, we retrieved a 12-lead ECG data set of a healthy control subject.

### Data usage statement

All raw ECG data can be downloaded from www.physionet.org^14^ as stated above. All other data (including all sound samples) are available as supplementary Files S1, S2 and S3. A detailed flowchart of the algorithm is available as supplementary File S4. All Matlab^®^ source codes used for this study are available under the MIT license (http://opensource.org/licenses/MIT) and can be accessed via the following DOI: [10.4119/unibi/2908653]. Also, we provide an implementation in for the open source platform SuperCollider that can be accessed via the following DOI: [10.4119/unibi/2908653]. All performance data collected during the data analysis by all observers are available as supplementary File S5.

### Computational implementation and hardware

The approaches described in the preceding sections have been implemented in Matlab^®^ (R2015b, Mathworks, Natick, MA, USA). All experiments were carried out on a standard computer workstation (2.2 GHz Intel Core i7, 16 GB RAM). All statistical calculations were carried out using Matlab^®^. Statistical error is given as mean ± standard deviation if not otherwise noted. To test for significance, we used one-tailed Student’s t-test. Sound samples were played on “Bose SoundLink Mini II” loudspeakers (Bose, Framingham, MA, USA). The entire code required to reproduce the experiments is freely available to the public (see “Data usage” section).

### Polyphonic ECG sonification

**Figure 1:**
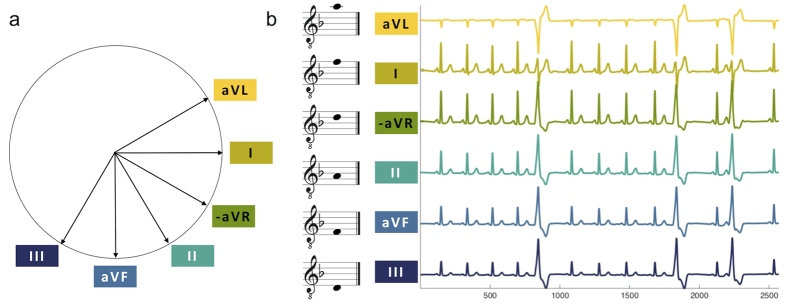
Principle of polyphonic sonification of multi-channel ECG data. **(a)** The Cabrera circle shows the direction of the ECG signal channels projected on a frontal plane through the human body. **(b)** In our technique, each of the six standard ECG channels is assigned a musical note so that the human auditory system can identify each channel even if multiple channels are played simultaneously. The sampling rate of the ECG signals was 257 Hz and the data shown in (**b**) correspond to 10 seconds.

The aim of our study was to develop and test a method for polyphonic sonification of pathological ECG signals. We used 6-channel ECG signals (Fig. 1a) and assigned each channel a note on the standard western chromatic musical scale (visualized in Fig. 1b as a musical note). The voltage of each ECG channel was mapped to the amplitude of the corresponding sound signal (and thus perceptually controls its loudness in a nonlinear way). Similar to Hermann *et al*.^17^, the voltage was furthermore continuously mapped to a frequency variation of 3% (i.e. half of a semi-tone) for each channel separately. In summary, higher (resp. lower) voltage manifest as louder and slightly up-pitched (resp. softer and slightly downpitched) notes, and the overall sonification is a continuous stream of six notes playing simultaneously.

For aesthetic reasons we selected the D minor scale (146.83 Hz, 174.61 Hz, 220.00 Hz, 293.67 Hz, 349.23 Hz, 440.00 Hz) over two octaves. In order to compensate for the unequal loudness at the different frequencies we linearly reduced the amplitude of the channels’ notes from 100% (for the lowest pitch) to 30% (for the highest pitch). While this is not exactly an equal loudness contour as suggested in the Robinson-Dadson curves, it is subjectively balanced. We also added a fixed set of harmonics 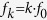 to each channel with k = 3, 4, 5 and amplitude as 15%, 5% and 5% of the fundamental frequency 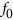). This results in a more complex timbre for the channels’ sound streams. Note that the 2^nd^ harmonic 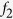 has been left out intentionally to diminish spectral confusion with the ECG channels 4–6, which are octave-shifted fundamentals of channels 1–3. We refer to this specific version of the parameter-mapping on time-variant oscillators as “polyphonic sonification”. A flowchart of the algorithm including relevant parameters is available in supplementary Figure S4.

The results of this sonification technique are available in the supplementary data: S1 (S1_normal_ECG.zip) contains a normal ECG of a healthy control sample. In S2 (S2_incremental_signal.zip), the channels from a pathological ECG are sonified incrementally, i.e. ECG lead III alone, then III and aVF, then III and aVF and II, etc. It can be heard that the individual channels can be identified even if they are played simultaneously.

### Data analysis

**Figure 2:**
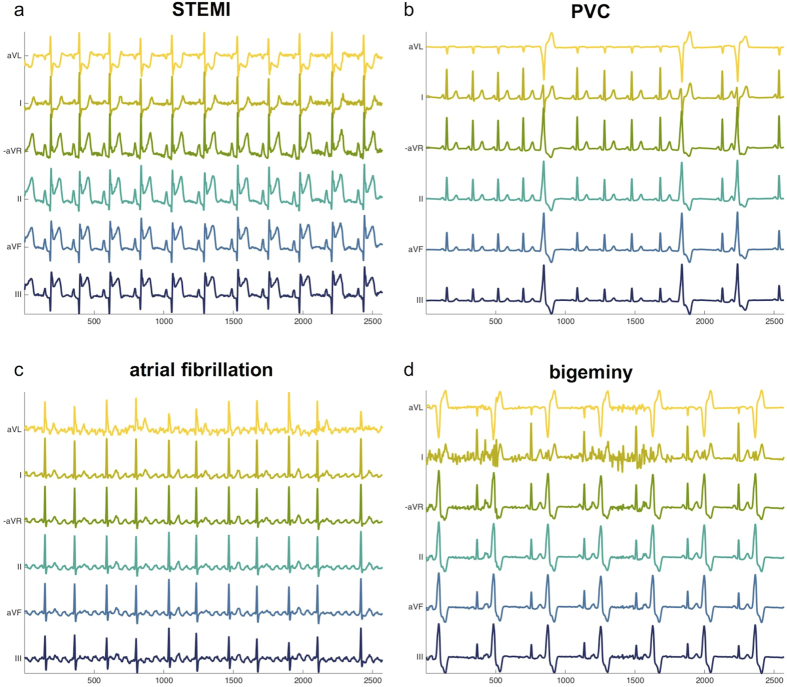
Pathological ECG samples used for auditory data analysis. (**a–d**) Sample ECG signals for clinically relevant cardiac pathologies. These samples were used for training of human observers. Subsequently, other samples were used to assess user performance in a blinded study. Channels mapped to lower frequencies are shown in blue/green hues while channels mapped to higher frequencies are shown in yellowish hues. The sampling rate of the ECG signals was 257/sec and the data correspond to 10 seconds.

After sonification, the data were analyzed by 22 blinded observers (one co-author of this paper [TK] and 21 other members of our departments and our professional environment). This was to test whether our technique can be used to distinguish clinically relevant cardiac pathologies. Observers who performed the data analysis belonged to any of the following three groups. Group 1: N = 10 medical students with completed cardiology course or young physicians in their first to third year of clinical practice (“cardio course completed”), Group 2: N = 7 medical students before completion of their cardiology course (“before cardiology course”), Group 3: N = 5 science students (undergraduate and graduate) with no formal training in cardiology whatsoever (“other science students”). We theoretically explained the method to all observers, demonstrated the incremental buildup of six channels to a polyphonic sound sample and successively played four pathological 10 second sound samples (one sample per target category, each sample played twice). During the demonstration of the sound samples, observers were visually shown the underlying data as presented in Fig. 2. Observers were not allowed to go back to the training examples during the testing session. Examples used during the training session were not re-used in the testing session. Then, we played 12 short (10 s) sound samples and asked the participants to classify each sample into exactly one of four categories. Examples for the four types of pathologies are depicted in Fig. 2 and can be listened to in the supplementary data S3 (S3_pathological_samples.zip).

## Results

### ECG datasets can be polyphonically sonified

In this study, we developed a new method to convert digital ECG signals to sound (“sonification”). We found that it is possible to process samples from a publicly available database and that the resulting sound is subjectively rated as pleasant (see supplementary File S2_incremental_signal.zip).

### Pathological ECG signals can be distinguished after sonification

**Figure 3:**
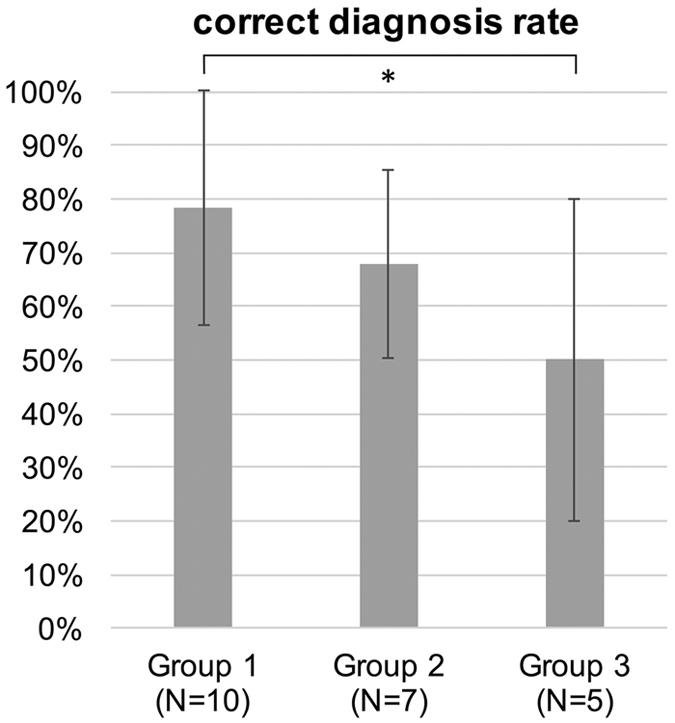
Group performance in blinded assessment of ECG signals. Average correct classification rate for each of the three observer groups.

**Figure 4:**
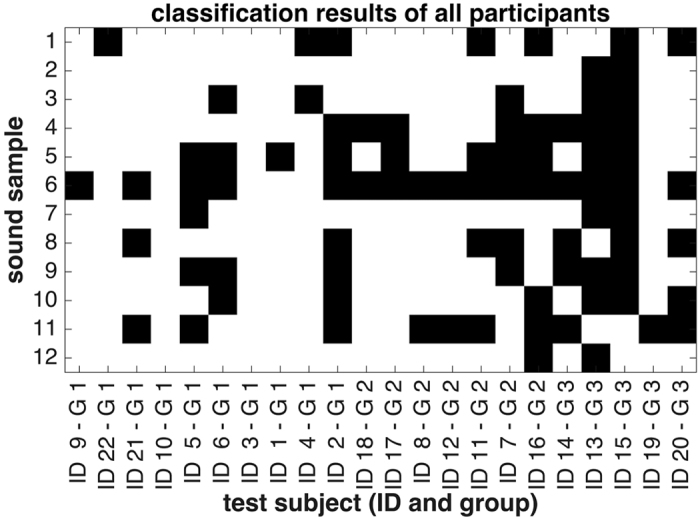
Classification performance of blinded observers. Data analysis results for 12 sound samples and 22 observers are shown. White cells show correct classification, black cells show wrong classification. Observers are ordered by their group (G) with G 1 = medical students with completed cardiology training or resident physicians; G 2 = medical students before their cardiology course; G 3 = science students without any formal training in cardiology.

To test human classification accuracy of sonified ECGs, sonified data were analyzed by N = 22 observers. After all observers had analyzed the data, we assessed whether sonified ECG signals could be used to distinguish clinically relevant cardiac pathologies. We found that in this analysis, there were marked differences between the three groups of observers (Fig. 3): Group 1 (medical students with completed cardiology training or resident physicians) scored highest (N = 10, average performance 78 ± 22%), followed by group 2 (medical students before their cardiology course, N = 7, 68 ± 18%). Group 3 (undergraduate or graduate science students without any formal training in cardiology) had the lowest scores (N = 5, 50 ± 30%). These values were all well above the expected baseline performance of 25% that corresponds to random guessing in a four-category classification experiment. We performed a univariate analysis (one-tailed student’s t-test) and found that Group 1 and Group 3 significantly differed (p = 0.028) in terms of classification performance. All other comparisons of groups were not significant (p > 0.05). 6 of 22 (27%) of all observers had a correct classification rate of over 90% (Fig. 4).

We also asked all observers whether they had been actively playing an instrument for three or more years at any time during their lives. In a univariate analysis of this variable, those N = 13 observers that had musical training achieved higher scores than the other group (74 ± 21% vs. 59 ± 28%). However, these differences were not statistically significant (p = 0.09, one-sided t-test).

### Premature ventricular contractions are most easily detected in sonified ECGs

**Figure 5:**
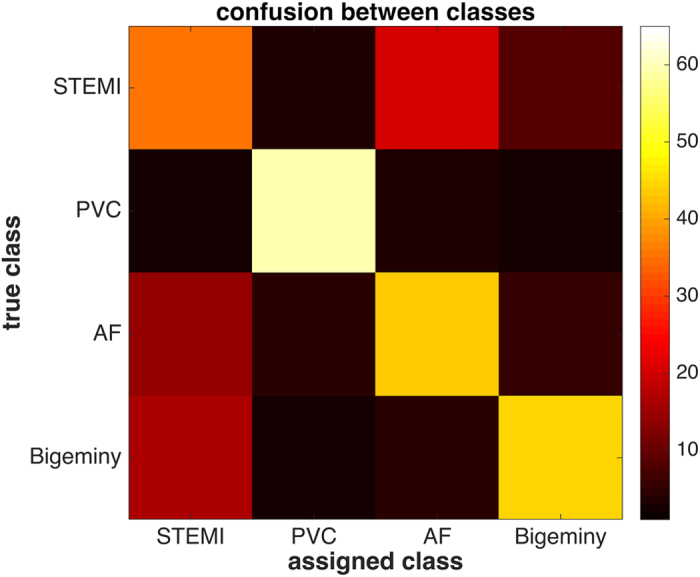
Confusion matrix of the classification. Classification performance is shown for all 22 observers for 264 classification tasks. Units on the color bar represent the number of samples. The vertical bar represents the true class, the horizontal bar represents the class assigned by human observers. Correctly classified samples are on the diagonal, while off-diagonal samples are not correctly classified. It can be seen that the class “PVC” showed the highest number of correct classifications (STEMI = ST-elevation myocardial infarction, PVC = premature ventricular contraction, A. Fib. = atrial fibrillation).

From the set of 264 data points in our experiments, we analyzed which type of ECG abnormality was most easily detected. We found that classification performance was best in the class “premature ventricular contraction” (PVC) with 89% correct classifications (see Fig. 5). This expected outcome can be attributed to the fact that it is the only of the 4 conditions where the rhythmical features deviate significantly. Generally, this underpins our assumption that rhythmical features and their deviation are a kind of structure that is easily perceived in an auditory display.

## Discussion

In this study, we demonstrate for the first time that minimally trained observers can successfully analyze sonified ECG data and detect clinically relevant pathological patterns. Although the training period was only approximately ten minutes, most observers were able to intuitively grasp the sonification technique and to successfully apply it to unknown samples. Classification performance was significantly better in those with formal training in cardiology compared to other observers. This shows that users who are already trained to visually detect abnormalities in ECG signals can make use of this ability in classifying sonified ECGs as well. Consequently, their mental representation of pathological ECG patterns is not restricted to visual patterns. Another interesting finding during our study was that 24% of all observers achieved very high classification accuracies (over 90%). These participants can serve as a proof of principle, showing that it is possible for human observers to reliably classify sonified pathological ECG patterns. During data analysis, several observers reported that they found the classification task to be easier towards the end of the analysis, suggesting a yet unexploited capacity of auditory learning and classification improvement with more extensive training or even longitudinal use. Further analyses of our data set showed that among the four selected ECG patterns, premature ventricular contractions were most easily detected. We attribute this to the fact that only in those ECG samples of our four conditions, a regular rhythm is disrupted by isolated events.

It should be noted that the present study has limited statistical power: 22 blinded observers analyzed the data and showed a good overall performance, in almost all cases well above the random guessing accuracy of 25%. Also, we detected differences between the groups, with the best performance among observers that were formally trained in cardiology. Still, to clearly demonstrate in which circumstances the sonification methods yields best results and which group of observers might benefit most, more research is needed. A first step would be the validation of our findings in a larger study with more different types of pathological ECG samples. We plan to optimize our method to render task-specific structures more salient, which can then evaluate refined sonification types against the actual baseline. It will also be interesting to investigate in how far a time-compression affects classification, assuming that a significant time reduction can be achieved for diagnosis.

Another interesting perspective is the combination of data sonification with data visualization. In our personal experience, simultaneous presentation of sonified and visualized ECG data allows a very efficient detection of abnormal signals. In the future, these synergies between visual and auditory data presentation should be further investigated.

Finally, concerning more precise differential diagnosis, more subtypes of pathological ECG patterns could be investigated such as anterior vs. posterior/inferior myocardial infarctions. Our study serves as a starting point for these inquiries because it demonstrates for the first time that humans are capable of making use of this type of data presentation in a clinical setting.

## Additional Information

**How to cite this article:** Kather, J.N. *et al*. Polyphonic sonification of electrocardiography signals for diagnosis of cardiac pathologies. *Sci. Rep.* **7**, 44549; doi: 10.1038/srep44549 (2017).

**Publisher's note:** Springer Nature remains neutral with regard to jurisdictional claims in published maps and institutional affiliations.

## Supplementary Material

Supplementary Information

Supplementary Material S1

Supplementary Material S2

Supplementary Material S3

Supplementary Material S5
