# Supplementary material for: Polyphonic sonification of electrocardiography signals for diagnosis of cardiac pathologies
Source: Sci Rep. 2017 Mar 20;7:44549. doi: 10.1038/srep44549 (PMC5357951; doi:10.1038/srep44549)
Supplement: Supplementary Information [file srep44549-s1.pdf]

Polyphonic sonification of electrocardiography signals for diagnosis of cardiac pathologies

SUPPLEMENTARY MATERIAL

Jakob Nikolas Kather[1,2], Thomas Hermann[3], Yannick Bukschat[1],

Tilmann Kramer[4], Lothar R. Schad[1], Frank Gerrit Zöllner[1]

[1] Computer Assisted Clinical Medicine, Medical Faculty Mannheim, Heidelberg University, Mannheim, Germany

[2] Department of Medical Oncology and Internal Medicine VI, National Center for Tumor Diseases, University Hospital Heidelberg, Heidelberg University, Heidelberg Germany

[3] Ambient Intelligence Group, Center of Excellence in Cognitive Interaction Technology (CITEC), Bielefeld University, Bielefeld, Germany

[3] Klinik III für Innere Medizin, Herzzentrum der Universität zu Köln, Cologne, Germany

S1 Supplementary Material: Normal ECG sample
S1_normal_ECG.zip

- Contains ECG data of a healthy control (original sound file in sub-folder "original", amplified sound file in sub-folder "amplified").
- *s0306lrem_output.wav* contains sound data for a six-channel data set, 10 seconds
- *s0306lrem_output.png* contains the corresponding visual data, 10 seconds, sampling rate of 1000/sec

S2 Supplement Material: Incremental signal
S2_incremental_signal.zip

- Contains an incremental number of channels from a pathological (STEMI) ECG data set (original sound file in sub-folder "original", amplified sound file in sub-folder "amplified").
- *incremental_01.wav* only channel III (lowest), 10 seconds
- *incremental_02.wav* channel III and aVF, 10 seconds
- *incremental_03.wav* channel III and aVF and II, 10 seconds
- *incremental_04.wav* channel III and aVF and II and -aVR, 10 seconds
- *incremental_05.wav* channels III through I, 10 seconds
- *incremental_06.wav* all channels, 10 seconds
- *incremental_visual.png* contains the corresponding visual data, 10 seconds, sampling rate of 257/sec

S3 Supplement Material: Pathological ECG samples
S3_pathological_samples.zip

- Contains sonified ECG data of four pathological samples, corresponding to Figure 2 in the main manuscript. All samples are 10 seconds in length (original sound file in sub-folder "original", amplified sound file in sub-folder "amplified").
- *Sample_I04m_STEMI_output.wav* ST-elevation myocardial infarction
- *Sample_I37m_PVC_output.wav* Premature ventricular contraction
- *Sample_I50m_AF_output.wav* Atrial fibrillation
- *Sample_I51m_Bigeminy_output.wav* Bigeminy

S4 Supplement Material: Flowchart of the algorithm

A detailed flowchart of the algorithm including all relevant parameters. This can be used to implement our proposed method in any programming language.

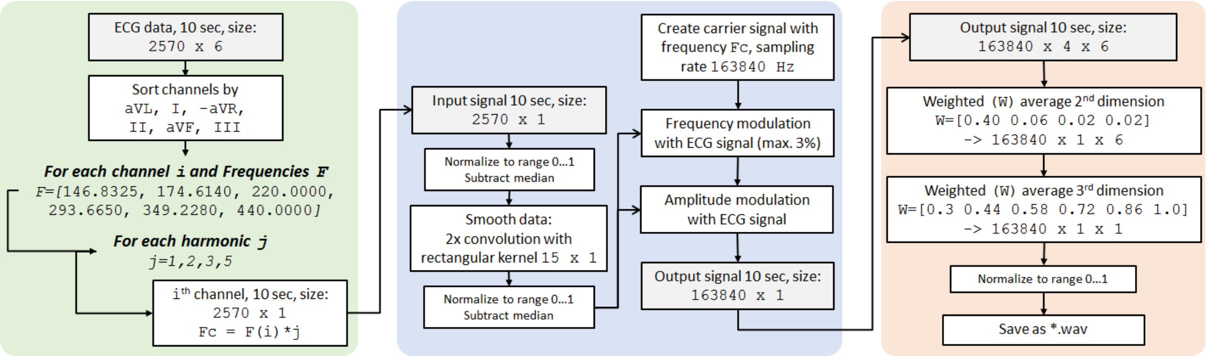

S5 Supplement Material: Observer performance during data analysis

S5_performance_data.xlsx

- Contains all results of the data analysis by 22 blinded observers in three groups. S1 to S12 refer to the ECG samples, the number in each cell in these columns shows the classification by the observer. Correct classifications are shown in green, errors are shown in red. “Instrument” denotes whether the observer had been actively playing an instrument for three or more years at any time during their life.
