# Supplementary figures and images for: Polyphonic sonification of electrocardiography signals for diagnosis of cardiac pathologies
Source: Sci Rep. 2017 Mar 20;7:44549. doi: 10.1038/srep44549 (PMC5357951; doi:10.1038/srep44549)

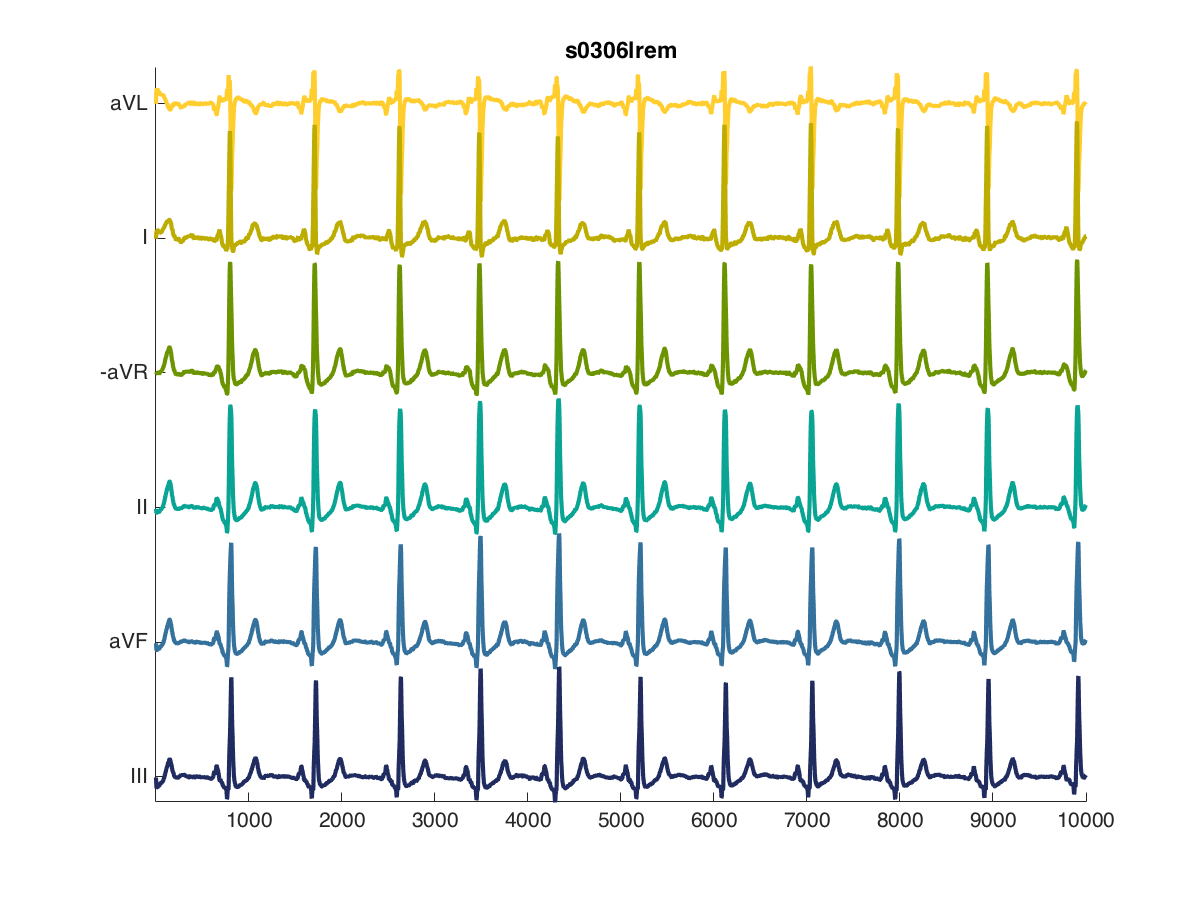

Supplement: Supplementary Material S1 [file srep44549-s2.zip › S1_normal_ECG/s0306lrem_output.png]
